# Anti-fibrotic effect of a selective estrogen receptor modulator in systemic sclerosis

**DOI:** 10.1186/s13287-022-02987-w

**Published:** 2022-07-15

**Authors:** Yena Kim, Yoojun Nam, Yeri Alice Rim, Ji Hyeon Ju

**Affiliations:** 1 Catholic iPSC Research Center, College of Medicine, The Catholic University of Korea, Seoul, South Korea; 2 YiPSCELL Inc., 47-3, Banpo-dearo 39-gil, Seocho-gu, Seoul, Republic of Korea; 3 Division of Rheumatology, Department of Internal Medicine, Seoul St. Mary’s Hospital, College of Medicine, The Catholic University of Korea, Seoul, 137-040 Republic of Korea

**Keywords:** Systemic sclerosis, Induced pluripotent stem cells, 3D skin organoid, Disease modeling, High-throughput screening, Drug repositioning, Raloxifene

## Abstract

**Background:**

The rarity of systemic sclerosis (SSc) has hampered the development of therapies for this intractable autoimmune disease. Induced pluripotent stem cell (iPSC) can be differentiated into the key disease-affected cells in vitro. The generation of patient-derived iPSCs has opened up possibilities for rare disease modeling. Since these cells can recapitulate the disease phenotypes of the cell in question, they are useful high-throughput platforms for screening for drugs that can reverse these abnormal phenotypes.

**Methods:**

SSc iPSC was generated from PBMC by Sendai virus. Human iPSC lines from SSc patients were differentiated into dermal fibroblasts and keratinocytes. The iPSC-derived differentiated cells from the SSc patients were used on high-throughput platforms to screen for FDA-approved drugs that could be effective treatments for SSc.

**Results:**

Skin organoids were generated from these cells exhibited fibrosis that resembled SSc skin. Screening of the 770-FDA-approved drug library showed that the anti-osteoporotic drug raloxifene reduced SSc iPSC-derived fibroblast proliferation and extracellular matrix production and skin fibrosis in organoids and bleomycin-induced SSc-model mice.

**Conclusions:**

This study reveals that a disease model of systemic sclerosis generated using iPSCs-derived skin organoid is a novel tool for in vitro and in vivo dermatologic research. Since raloxifene and bazedoxifene are well-tolerated anti-osteoporotic drugs, our findings suggest that selective estrogen receptor modulator (SERM)-class drugs could treat SSc fibrosis.

**Supplementary Information:**

The online version contains supplementary material available at 10.1186/s13287-022-02987-w.

## Background

Scleroderma (SSc) is heterogeneous disease, and the pathogenesis is characterized by three hallmarks of small vessel vasculopathy, autoantibodies, and fibroblast dysfunction resulting in increased deposition of extracellular matrix [1, 2]. SSc patients exhibit progressive fibrosis and vascular abnormalities in the skin and multiple internal organs that can lead to fatal systemic complications [3, 4]. There are two major classifications of scleroderma: localized sclerosis and systemic sclerosis. Localized sclerosis is usually founded in only a few places on the skin or muscles, and rarely spread elsewhere. Localized sclerosis is rarely developed to systemic scleroderma. Systemic sclerosis is affected the connective tissue in many parts of the body. Systemic scleroderma is involved the skin, esophagus, gastrointestinal tract, lung, kidney, heart, and other internal organs and affected blood vessels, muscle, and joint. Systemic sclerosis is recognized of two major types as diffuse scleroderma and limited scleroderma. In diffuse scleroderma, skin thickness and fibrosis are occurred more rapidly and spread to other skin area than limited scleroderma [5]. The exact pathophysiological mechanism of systemic sclerosis is not known, and the treatment of SSc continues to present a major challenge [6, 7], partly because of its heterogeneity and partly because it is an orphan disease and patient-derived biomaterials are scarce. The latter has greatly hampered research on SSc pathophysiology and the development of effective treatments.

A promising strategy for overcoming the biomaterial scarcity in orphan diseases is to generate induced pluripotent stem cells (iPSCs) by transducing the somatic cells of patients with reprogramming factor genes (e.g., OCT4, SOX2, KLF4, and c-MYC) [8–10]. The resulting iPSCs can be differentiated into the key disease-affected cells in vitro. Since these cells can recapitulate the disease phenotypes of the cell in question, they are useful high-throughput platforms for screening for drugs that can reverse these abnormal phenotypes.

Organoid is a 3D multicellular in vitro tissue construct that mimics its corresponding in vivo organ, such that can be derived from adult cells or pluripotent stem cells [11]. Organoid can be used to study aspects of that organ in the culture dish [12, 13]. Thus, we generated iPSC lines from peripheral blood mononuclear cells (PBMCs) from SSc patients and healthy controls and differentiated them into skin cells, namely keratinocytes and fibroblasts. We generated the 3D skin using iPSC-derived keratinocytes and fibroblasts and that skin mimics its corresponding in vivo skin. When these cells were co-cultured in a three-dimensional layered system, they formed skin organoids, whether the skin organoids recapitulated the SSc-skin phenotype, both in vitro and when xenografted onto immunodeficient mice, was assessed.

We also used the iPSC-derived fibroblasts from the SSc patients as high-throughput platforms to screen for FDA-approved drugs that could be effective treatments for SSc. The iPSC-derived fibroblasts were used because the main therapeutic target in SSc is its rampant fibrosis, and therapies that can halt fibrosis in SSc are not yet available [14]. Our experiments showed that raloxifene, a selective estrogen receptor antagonist, may be a candidate treatment for SSc. Thus, the SSc iPSC-based platform was useful for disease modeling, drug screening, and drug repositioning.

## Materials and methods

### PBMC isolation

Blood was collected into heparin tubes, diluted with phosphate-buffered saline (PBS), and centrifuged through a Ficoll gradient (GE Healthcare) for 30 min at 850* g*. The PBMCs were washed, resuspended in StemSpan medium (STEMCELL Technologies) supplemented with CC110 cytokine cocktail (STEMCELL Technologies), and cultured for 5 days at 37℃ in 5% CO_2_ before being reprogrammed. PBMCs were collected from five SSc patients subjects, and the clinical information of the patients is shown in Table 1.

**Table 1:** Clinical information of systemic sclerosis patients

No	Sex	Age range	Disease duration, years	Skin involvement	ILD	PAH	GI involvement	ANA	Anti-centromere	Anti-scl70
1	2	40–49	20	Limited	Yes, NSIP	No	GERD	1:80, nucleolar	Negative	Negative
2	1	40–49	13	Diffuse	Yes, NSIP + UIP	Yes	Recurrent paralytic ileus	1:1280, speckled	Negative	Negative
3	1	50–59	2	Diffuse	Yes, UIP	No	GERD	1:1280, cytoplasmic	Negative	Positive
4	2	60–69	5	Diffuse	Yes, NSIP	No	GERD	1:80, speckled	Negative	Negative
5	1	50–59	16	Diffuse	No	No	GERD, scleroderma esophagus	1:640, speckled	Negative	Negative

1, Female; 2, Male; ILD, interstitial lung disease; PAH, pulmonary artery hypertension; ANA, antinuclear antibody; NSIP, nonspecific interstitial pneumonia; UIP, usual interstitial pneumonia; GERD, gastroesophageal reflux disease

### Generation of iPSCs from PBMC by Sendai virus-based reprogramming

Human iPSCs were generated from the PBMCs by using the CytoTune-iPS Sendai Reprogramming kit (Life Technologies) as described previously [15, 16]. Thus, Sendai viruses that expressed the four Yamanaka factors were added to the PBMCs at a multiplicity of infection of 7.5 per 3 × 10^5^ cells. After transduction, the PBMCs were centrifuged at 1160* g* for 30 min and then incubated at 37℃ in 5% CO_2_. The next day, the cells were transferred to a vitronectin-coated plate (Thermo Fisher Scientific) and settled by centrifugation for 10 min at 1160* g*. These reprogrammed iPSCs were maintained in vitronectin-coated culture dishes containing TeSR-E8 medium (STEMCELL Technologies) that was changed daily. Cell morphology was examined by Leica microscopy.

### Alkaline-phosphatase and immunocytochemical staining of iPSCs

The iPSCs were expanded for 5–7 days with daily medium changes. The resulting large iPSC colonies were subjected to alkaline-phosphatase staining and immunocytochemical analysis. For alkaline-phosphatase staining, the cells were fixed in 4% paraformaldehyde for 1–2 min, after which the staining solution (Fast Red Violet, Naphthol AS-BI phosphate solution, and water in a 2:1:1 ratio) was added. After incubation in the dark for 15 min, the stained colonies were examined by bright-field microscopy. For immunocytochemical staining, the iPSCs were washed with PBS and then fixed with 4% paraformaldehyde for 30 min. The cells were permeabilized with 0.1% Triton X-100 for 10 min, blocked for 30 min at room temperature in PBS containing 2% bovine serum albumin (Sigma-Aldrich) (PBA), and stained at room temperature for 2 h with primary antibodies diluted in PBA as follows: Oct4 (Santa Cruz Biotechnology, 1:100 dilution), Klf4 (Abcam, 1:250), Sox2 (BioLegend, 1:100), TRA-1–60 (EMD Millipore, 1:100), TRA-1–81 (EMD Millipore, 1:100), and SSEA4 (EMD Millipore, 1:200). The cells were then treated with the secondary Alexa Fluor 594- and 488-conjugated secondary antibodies (Life Technologies) diluted 1:400 in PBA. After 1 h incubation at room temperature, the cells were washed, and counterstaining was conducted with DAPI (blue). The cells were mounted by using ProLong Antifade mounting reagent (Thermo Fisher Scientific) and analyzed by Leica immunofluorescence microscopy.

### Teratoma formation

All procedures involving animals were conducted in accordance with the Laboratory Animals Welfare Act, the Guide for the Care and Use of Laboratory Animals, and the Guidelines and Policies for Rodent Experimentation of the Institutional Animal Care and Use Committee of the College of Medicine of the Catholic University of Korea. The study protocol was approved by the Institutional Review Board of the Catholic University of Korea (CUMC-2016-0291-02). To further assess iPSC pluripotency, their ability to form teratomas was examined [17–19]. Pluripotency of the cell lines is confirmed by whether the teratoma contains tissues derived from each of the embryonic germ layers: endoderm, mesoderm, and ectoderm. Thus, the iPSC colonies were dissociated and 1 × 10^6^ cells were resuspended in a 1:1 solution of Matrigel (BD Biosciences) and Dulbecco’s Modified Eagle Medium (DMEM, Gibco) mixed 1:1 with F12 medium (Gibco). The iPSCs were then injected into the testis capsule of immunodeficient mice (NOD/SCID, Jackson). After 6–12 weeks, the tumor tissue was excised and subjected to H&E staining and histology to assess whether all three germ layers were present. Histological analysis was examined by Leica microscopy.

### Differentiation of iPSCs into keratinocytes and fibroblasts

The iPSCs were induced to differentiate into keratinocytes and fibroblasts as described previously [20–24]. In our previous study, we established the protocol for differentiating iPSCs into fibroblast and keratinocyte [25, 26]. Briefly, the iPSCs were first induced to form EBs by using the hanging drop method. This method ensures uniform and well-controlled differentiation. The iPSCs were differentiated into keratinocytes by attaching the EBs to a plate coated with type IV collagen (Santa Cruz Biotechnology) on day 0. Over the next 21 days, the EBs were cultured sequentially with three keratinocyte differentiation media on days 1–7, 8–11, and 12–30, respectively. Keratinocyte differentiation medium 1 was a 3:1 mixture of DMEM and F12 medium supplemented with 2% FBS, 0.3 mmol/l L-ascorbic acid, 5 μg/ml insulin, 24 μg/ml adenine, 1 μg/ml retinoic acid (Sigma-Aldrich), 25 ng/ml BMP4, and 20 ng/ml EGF. Keratinocyte differentiation medium 2 consisted of defined keratinocyte serum-free medium (Gibco) without FBS but supplemented with all the other supplements in keratinocyte differentiation medium 1 (the concentrations were all the same except adenine was present at 10 μg/ml). Keratinocyte differentiation medium 3 consisted of a 1:1 mixture of keratinocyte serum-free medium (Gibco) and defined keratinocyte serum-free medium supplemented with 25 ng/ml BMP4 and 20 ng/ml EGF.

To induce iPSCs to differentiate into fibroblasts, the EBs were attached to a Matrigel-coated plate on day 0. The cells were then incubated sequentially in three fibroblast differentiation media on days 1–3, 4–6, and 7–14, respectively. Fibroblast differentiation medium 1 consisted of a 3:1 mixture of DMEM and F12 medium supplemented with 5% fetal bovine serum (FBS), 5 μg/ml insulin, 24 μg/ml adenine, and 10 ng/ml epidermal growth factor (EGF; R&D). On days, 4–6 fibroblast differentiation medium 1 was supplemented with 6.5 ng/ml bone morphogenetic protein-4 (BMP4; R&D). Fibroblast differentiation medium 2 consisted of a 1:1 mixture of DMEM and F12 medium supplemented with 5% FBS and 1% non-essential amino acids on day 7–14. On days 14 and 21, the cells were passaged onto non-coated and type I collagen-coated dishes (BD Biosciences), respectively, in fibroblast differentiation medium 1. For each experiment, the eight iPSC cell lines were subjected to the differentiation process five times.

### RNA isolation and qRT-PCR of cells differentiated from iPSCs

Total mRNA was extracted from the iPSC-derived fibroblasts and keratinocytes by using Trizol (Life Technologies) and cDNA was synthesized by using a RevertAid™ First Strand cDNA Synthesis kit (Thermo Fisher Scientific). RT-PCRs were performed with the LightCycler® 480 instrument (Roshe), the SYBR Green Real-time PCR Master Mix (Roshe), and the OCT4, CD44, COL1A1, COL1A2, COL3A1, vimentin, PAX6, SOX1, Np63, KRT5, and KRT14 primer sequences shown in Table 2. The gene expression levels were normalized to GAPDH expression levels.

**Table 2:** Sequence of primers used for quantitative RT-PCR

Target name	Direction	Primer sequence	Size
hOCT4	Forward	ACCCCTGGTGCCGTGAA	190
	Reverse	GGCTGAATACCTTCCCAAATA	
hCD44	Forward	AAGGTGGAGCAAACACAACC	151
	Reverse	AGCTTTTTCTTCTGCCCACA	
hCOL1A1	Forward	CCCCTGGAAAGAATGGAGATG	148
	Reverse	TCCAAACCACTGAAACCTCTG	
hCOL1A2	Forward	GGATGAGGAGACTGGCAACC	77
	Reverse	TGCCCTCAGCAACAAGTTCA	
hCOL3A1	Forward	CGCCCTCCTAATGGTCAAGG	161
	Reverse	TTCTGAGGACCAGTAGGGCA	
hVimentin	Forward	GAGAACTTTGCCGTTGAAGC	170
	Reverse	TCCAGCAGCTTCCTGTAGGT	
hPAX6	Forward	GTCCATCTTTGCTTGGGAAA	110
	Reverse	TAGCCAGGTTGCGAAGAACT	
hSOX1	Forward	CACAACTCGGAGATCAGCAA	133
	Reverse	GGTACTTGTAATCCGGGTGC	
hNp63	Forward	GGAAAACAATGCCCAGACTC	294
	Reverse	GTGGAATACGTCCAGGTGGC	
hKRT5	Forward	ACCGTTCCTGGGTAACAGAGCCAC	198
	Reverse	GCGGGAGACAGACGGGGTGATG	
hKRT14	Forward	GCAGTCATCCAGAGATGTGACC	181
	Reverse	GGGATCTTCCAGTGGGATCT	
hGAPDH	Forward	ACCCACTCCTCCACCTTTGA	110
	Reverse	CTGTTGCTGTAGCCAAATTCGT	
mCOL1A1	Forward	GCAACAGTCGCTTCACCTACA	138
	Reverse	CAATGTCCAAGGGAGCCACAT	
mCOL3A1	Forward	TGAGCGTGGCTATTCCTTCGT	76
	Reverse	GCCGTGGCCATCTCATTTTCAA	
mACTA2	Forward	GTTCTAGAGGATGGCTGTACTA	108
	Reverse	TTGCCTTGCGTGTTTGATATTC	
mGAPDH	Forward	ACCCCAGCAAGGACACTGAGCAAG	92
	Reverse	TGGGGGTCTGGGATGGAAATTGTG	

### Immunocytochemical staining of cells differentiated from iPSCs

The differentiated cells were fixed with 4% paraformaldehyde, permeabilized by using 0.1% Triton X-100, stained with primary antibodies against Keratin14 (Abcam, 1:200), Np63 (Abcam, 1:100), vimentin (Abcam, 1:200) or fibronectin (Abcam, 1:200) for 1 h, and then incubated with Alexa488/594-conjugated goat anti-mouse or goat anti-rabbit secondary antibodies. Counterstaining was conducted with DAPI (blue). The stained cells were observed by immunofluorescence microscopy.

### Flow cytometric analysis of cells differentiated from iPSCs

The differentiated cells were fixed by using a Foxp3 Transcription Factor Staining Buffer Kit (Affymetrix) and then stained with antibodies against CD73, CD105, CD45, and CD34. The stained cells were then analyzed by using an LSR Fortessa Cell Analyzer (BE Biosciences). The data were analyzed by using Flowjo V10 single-cell analysis software (TreeStar Inc).

### Proliferation assay of iPSC-derived fibroblasts

The iPSCs before and after their differentiation into fibroblasts were seeded onto 96-well plates, incubated for 24 h, and then incubated for 1–4 h with 10 μl/well of Cell Counting Kit-8 solution (Dojindo). Absorbance at 450 nm was measured by using a microplate reader (VersaMax).

### Western blot analysis of iPSC-derived fibroblasts

Cellular protein was harvested by using RIPA buffer (Sigma) and quantified by using a BCA protein assay (GenDEPOT). Equal protein amounts were resolved by 10% SDS-PAGE and transferred to polyvinylidene difluoride membranes (Amersham Pharmacia Biotech). The membranes were incubated overnight with specific antibodies against α-SMA (Abcam), pSMAD2 (Thermofisher), SMAD2 (Thermofisher) and anti-GAPDH (Abcam). The next day, the membranes were washed, incubated with a peroxidase-linked IgG (Abcam), and visualized by using an ECL kit (WESTSAVE Gold).

### Hydroxyproline assay of iPSC-derived fibroblasts

Measurement of collagen concentrations was performed using hydroxyproline assay kit (Sigma-Aldrich). Homogenize cells or supernatant were mixed with hydrochloric acid and incubated at 120 ℃ for 3 h. Add 5 mg of activated charcoal and centrifuge at 13,000*g* for 2 min. Transfer supernatant to a 96 well plate and dry all wells under vacuum. Add the Chloramine T/Oxidation Buffer mixture to each well and incubate at RT for 5 minutes. After 5 minutes, add the Diluted DMAB Reagent to each sample and incubated for 90 min at 60 ℃. Absorbance at 560 nm was measured by using a microplate reader (VersaMax).

### Three-dimensional skin organoid culture

Differentiated fibroblasts were resuspended in neutralized type 1 collagen solution (BD Biosciences), and 2 × 10^5^ cells were added to each insert of a Transwell plate (Corning) with fibroblast differentiation medium 1. After 5–7 days of incubation, 1 × 10^6^ keratinocytes were seeded onto the fibroblast layer in low-calcium epithelial medium for 2 days. The bilayer was then submerged for 2 days in normal calcium medium. After another 4 days, the normal calcium medium was only added to the bottom of the insert so that an air/liquid interface was created.

### Histological analysis of the skin organoids

The organoids were fixed in 4% paraformaldehyde for 1 h at room temperature and then dehydrated and cleared with graded ethanol and xylene. After paraffin infiltration and embedding in paraffin blocks, the organoids were sectioned into 8 μm slices by using a microtome. The skin area and thickness of the organoids were determined by image j program. Quantify was performed according to the manufacturer’s instruction.

### Phospho-Kinase Array

Proteome Profiler human Phospho-Kinase Array Kit (R&D Systems) was performed according to the manufacturer’s instructions for analyzing proteomics analysis. Briefly, phospho-kinase array detects relative phosphorylation levels of individual analytes. Parts A and B of each array were incubated with 200 μg of cell lysate. Each spotted in duplicate with antibodies against 37 different kinases.

### Xenografting of iPSC-derived skin organoids in mice

All procedures involving animals were conducted in accordance with the Laboratory Animals Welfare Act, the Guide for the Care and Use of Laboratory Animals, and the Guidelines and Policies for Rodent Experimentation of the Institutional Animal Care and Use Committee of the College of Medicine of the Catholic University of Korea. The study protocol was approved by the Institutional Review Board of the Catholic University of Korea (CUMC-2017-0150-01). About 1 × 2 cm of the dorsal skin of male NOD/SCID mice (*n* = 5 per group, 6 weeks old, Jackson Laboratories) was removed and the organoids from SSc patients and healthy controls were placed in the defects and sutured with silk sutures by using the tie-over dressing method. After 2 weeks, the mice were killed and their organoids were subjected to histology using Leica microscopy.

### Primary screening of the FDA-approved drug library

For the primary screen, iPSC-derived fibroblasts from SSc patients were seeded into 96-well plates at 1 × 10^4^ cells/well, incubated for 24 h in fibroblast differentiation medium 1, and then treated with each FDA-approved drug (ENZO life Science) or the vehicle control (DMSO) for 1 h. Cell proliferation was detected by using Cell Counting Kit-8 (Dojindo). The drugs selected in the primary screen were then assessed for their ability to reduce the total collagen content by using the hydroxyproline assay.

### Treatment of mice with bleomycin-induced SSc with raloxifene

All procedures involving animals were conducted in accordance with the Laboratory Animals Welfare Act, the Guide for the Care and Use of Laboratory Animals, and the Guidelines and Policies for Rodent Experimentation of the Institutional Animal Care and Use Committee of the College of Medicine of the Catholic University of Korea. The study protocol was approved by the Institutional Review Board of the Catholic University of Korea (CUMC-2017-0128-05). To generate mice with bleomycin-induced SSc, C57BL/6 mice were injected subcutaneously with 1 μg bleomycin (NIPPON KAYAKU) every day for 3 weeks. The model was generated by daily subcutaneous bleomycin injections. Starting 3 days after the bleomycin injections began, the mice were also injected subcutaneously with 10 mg/kg raloxifene (Takeda). Starting three days later, the mice were also treated with daily subcutaneous injections of raloxifene. The mice were killed. The double injections were continued for the remaining 21 days of the experiment. The skin was subjected to histology and western blot analysis of fibrosis markers expression.

### Histological analysis

Skin was fixed in 4% paraformaldehyde at room temperature, and then dehydrated and cleared using graded ethanol and xylene. After paraffin infiltration and embedding, paraffin blocks of the skin were sectioned at a thickness of 8 μm using a microtome. Slides were dried for 60 min at 60 °C and deparaffinized by two incubations with xylene. Sections were rehydrated using a decreasing sequential ethanol series and rinsed under tap water for 5 min. For hematoxylin and eosin (H&E) staining, sections were incubated in Harris’ hematoxylin solution for 10 min, washed with 1% HCl-ethanol, neutralized in 0.2% ammonia water, and counterstained with eosin for 1 min. For Picrosirius Red (PSR) staining, slides were incubated in PSR solution for 1 h and washed with acetic acid. For Masson’s trichrome staining, slides were re-fixed in Bouin’s solution overnight at room temperature and incubated in Weigert’s hematoxylin for 10 min, Biebrich scarlet-acid fuchsin for 5 min, and a mixture of phosphotungstic acid, phosphomolybdic acid, and distilled water (1:1:2) for 10 min. Thereafter, slides were directly transferred to 2% aniline blue, incubated for 5 min, washed with 1% acetic acid, and then incubated in an increasing sequential ethanol series. Ethanol was cleared using xylene and slides were mounted using VectaMount permanent mounting medium (Vector laboratories Burlingame, CA, USA). Staining was examined underneath a bright-field microscope.

### Statistical analyses

The results are expressed as mean and standard error of the mean. Error bars indicate the standard error of the mean. Groups were compared by using Student’s *t* test and calculating the one-tailed *p* value (*p < 0.05, **p < 0.01, ***p < 0.001 indicated statistical significance). All statistical analyses were performed by using GraphPad Prism 9 (GraphPad Software).

## Results

### Generation of iPSCs from SSc patients and control subjects

**Fig. 1:**
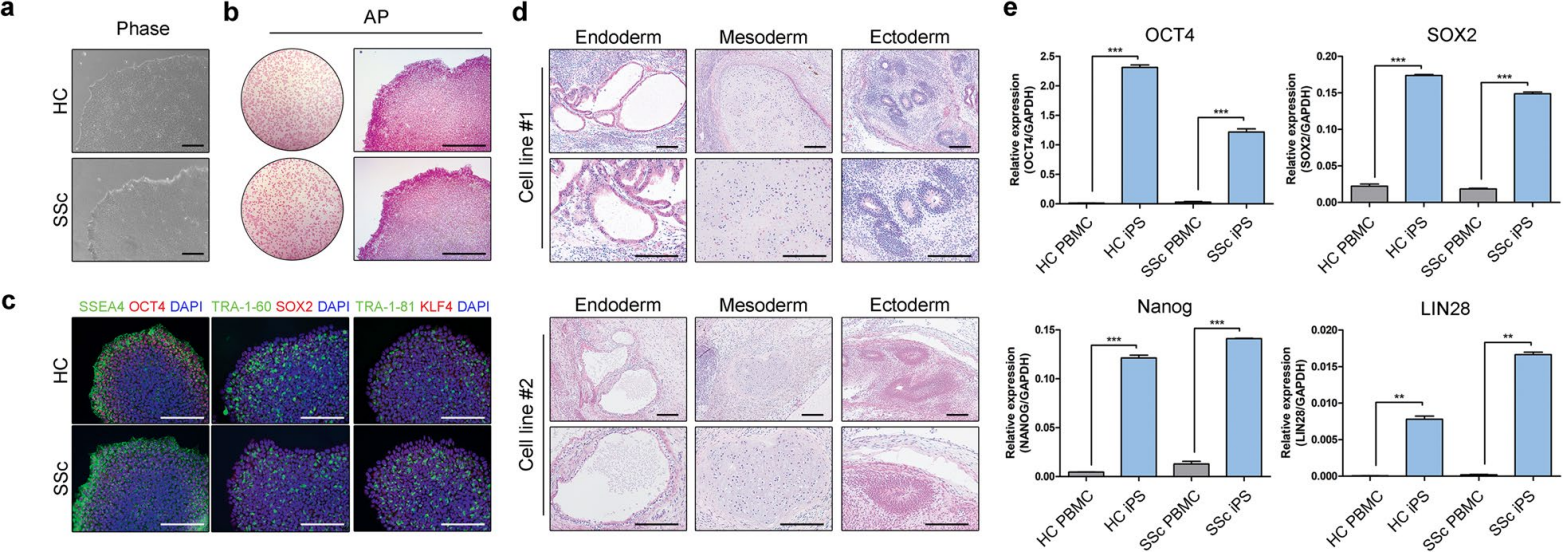
Characterization of induced pluripotent stem cells (iPSCs) from patients with systemic sclerosis (SSc) and healthy controls. **a** Morphology of iPSCs, as determined by Leica microscopy. Scale bars, 200 μm. **b** Alkaline phosphatase staining of iPSCs. Scale bars, 200 μm. **c** Immunocytochemical analysis of iPSC expression of the pluripotency-marker proteins Oct4, SSEA4, Sox2, TRA-1-60, Klf4, and TRA-1-81. Scale bars, 200 μm. **d** H&E staining of the teratomas generated from the iPSCs. **e** RT-PCR analysis of iPSC expression of the pluripotency-marker genes OCT4, SOX2, NANOG, and LIN28. All graphs show the mean and standard error of the mean of five replicates of each of the iPSC lines (*p < 0.05, **p < 0.01, ***p < 0.001, as determined by Student’s *t *test)

Stable iPSC lines were generated from five SSc patients and three healthy-control subjects by reprogramming their PBMCs with Sendai viruses containing the four Yamanaka factors (Klf4, Oct3/4, Sox2, and c-Myc) and then passaging the virus-transduced cells. The clinical information of the SSc patients is shown in Table 1. Regardless of whether they were from patients or healthy controls, the iPSC lines resembled human embryonic-stem cells in terms of morphology (Fig. 1a and Additional file 1: Fig. S1a). They also expressed alkaline phosphatase (Fig. 1b and Additional file 1: Fig. S1b) and expressed multiple pluripotency-marker proteins, namely Oct4, SSEA4, Sox2, TRA-1-60, Klf4, and TRA-1-81 (Fig. 1c and Additional file 1: Fig. S1c). To confirm the pluripotency of the iPSCs, they were injected into the testicular capsule of immunodeficient mice to determine whether they could induce teratomas [17–19]. Indeed, SSc iPSCs proliferated and differentiated into all three germ layers (Fig. 1d). Moreover, they expressed several pluripotent-marker genes (OCT4, SOX2, NANOG, and LIN28) (Fig. 1e and Additional file 1: Fig. S1d). Thus, iPSCs were successfully generated from the PBMCs of the SSc patients and the healthy controls.

### Generation of iPSC-derived keratinocytes and fibroblasts

The iPSC lines were induced to form embryoid bodies (EBs), after which the EBs were transferred to type IV collagen- or Matrigel-coated dishes and subjected to culture conditions that caused their outgrowing cells to differentiate into keratinocytes or fibroblasts [20, 22, 24, 27].

**Fig. 2:**
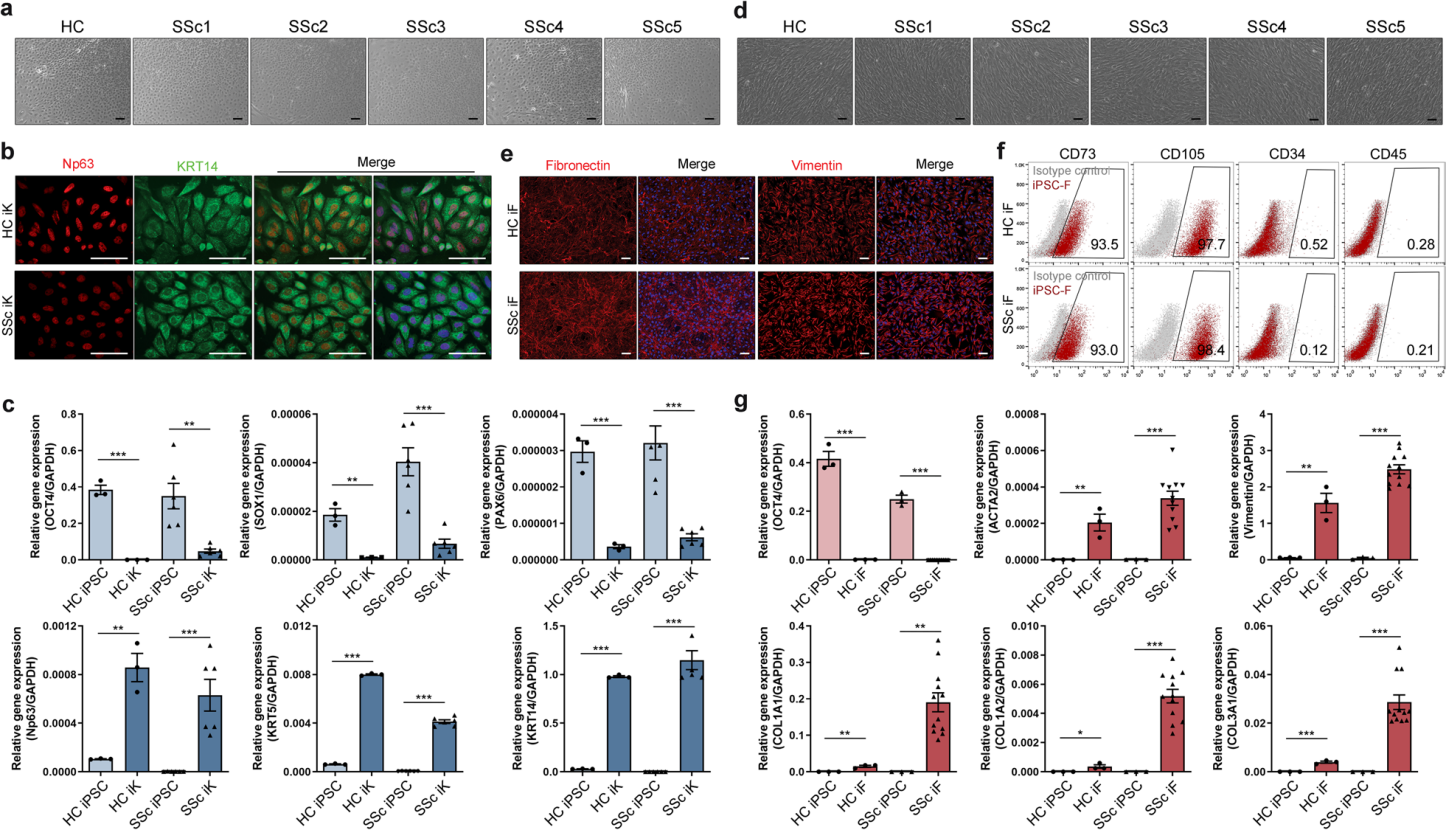
Differentiation of induced pluripotent stem cells (iPSCs) into fibroblasts and keratinocytes. **a**–**c** Characteristics of the iPSC-derived keratinocytes on day 21. **a** Morphology, as determined by Leica microscopy. Scale bars, 100 μm. **b** Immunocytochemical analysis of Np63 (red) and Keratin14 (green), together with DAPI staining (blue). Scale bars, 100 μm. **c** Gene expression of an iPSC marker (OCT4), neuroectoderm markers (PAX6 and SOX1), and keratinocyte markers (Np63, KRT5, and KRT14). **d**–**f** Characteristics of the iPSC-derived fibroblasts on day 21. **d** Morphology, as determined by Leica microscopy. Scale bars, 100 μm. **e** Immunocytochemical analysis of the fibroblast-marker proteins fibronectin (red) and vimentin (red), together with DAPI staining (blue). Scale bars, 100 μm. **f** Flow cytometric analysis of CD34, CD45, CD73, and CD105 expression. All graphs show the mean and standard error of the mean. **g** Expression of the pluripotency-marker gene OCT4 and the fibroblast-marker genes COL1A1, COL1A2, COL3A1, ACTA2, and vimentin (*p < 0.05, **p < 0.01, ***p < 0.001, as determined by Student’s *t-*test)

On day 14, the iPSC-derived keratinocytes had a primary keratinocyte-like cobblestone morphology when cultured on type IV collagen-coated dishes (Fig. 2a). These cells expressed the keratinocyte-marker Np63 and KRT14 protein (Fig. 2b). Their expression of OCT4, the pluripotency-marker gene, and the neuroectoderm-marker genes PAX6 and SOX1 was decreased compared to iPSCs (Fig. 2c). Thus, the iPSC-derived keratinocytes lacked the characteristics of iPSCs and did not differentiate along the neuroectoderm lineage. These cells also expressed the keratinocyte-marker genes Np63, KRT5, and KRT14 (Fig. 2c).

On day 21, the iPSC-derived fibroblasts had a similar morphology as 3T3 cells, which is an established fibroblast-cell line [25]. SSc iPSC-derived fibroblasts had a similar morphology as flat, elongated and spindle-shaped (Fig. 2d). They expressed fibronectin and vimentin protein, which are well-known markers of fibroblasts (Fig. 2e). Furthermore, flow cytometric analyses showed that their expression of the hematopoietic stem cell markers CD34 and CD45 was lowed and their expression of the fibroblast-surface markers CD73 and CD105 was high (Fig. 2f). They also expressed OCT4, a pluripotency-marker gene, at lower levels than undifferentiated iPSCs and expressed numerous fibroblast-marker genes (i.e., COL1A1, COL1A2, COL3A1, ACTA2 and vimentin). Especially, the gene expression was increased in SSc iPSC-derived fibroblast than health-control (Fig. 2g). Thus, these results confirmed that iPSC-derived keratinocytes and fibroblasts resembled primary cell lines in terms of gene expression, protein expression and morphology.

### In vitro and in vivo modeling of SSc disease by using differentiated iPSC-derived cells

**Fig. 3:**
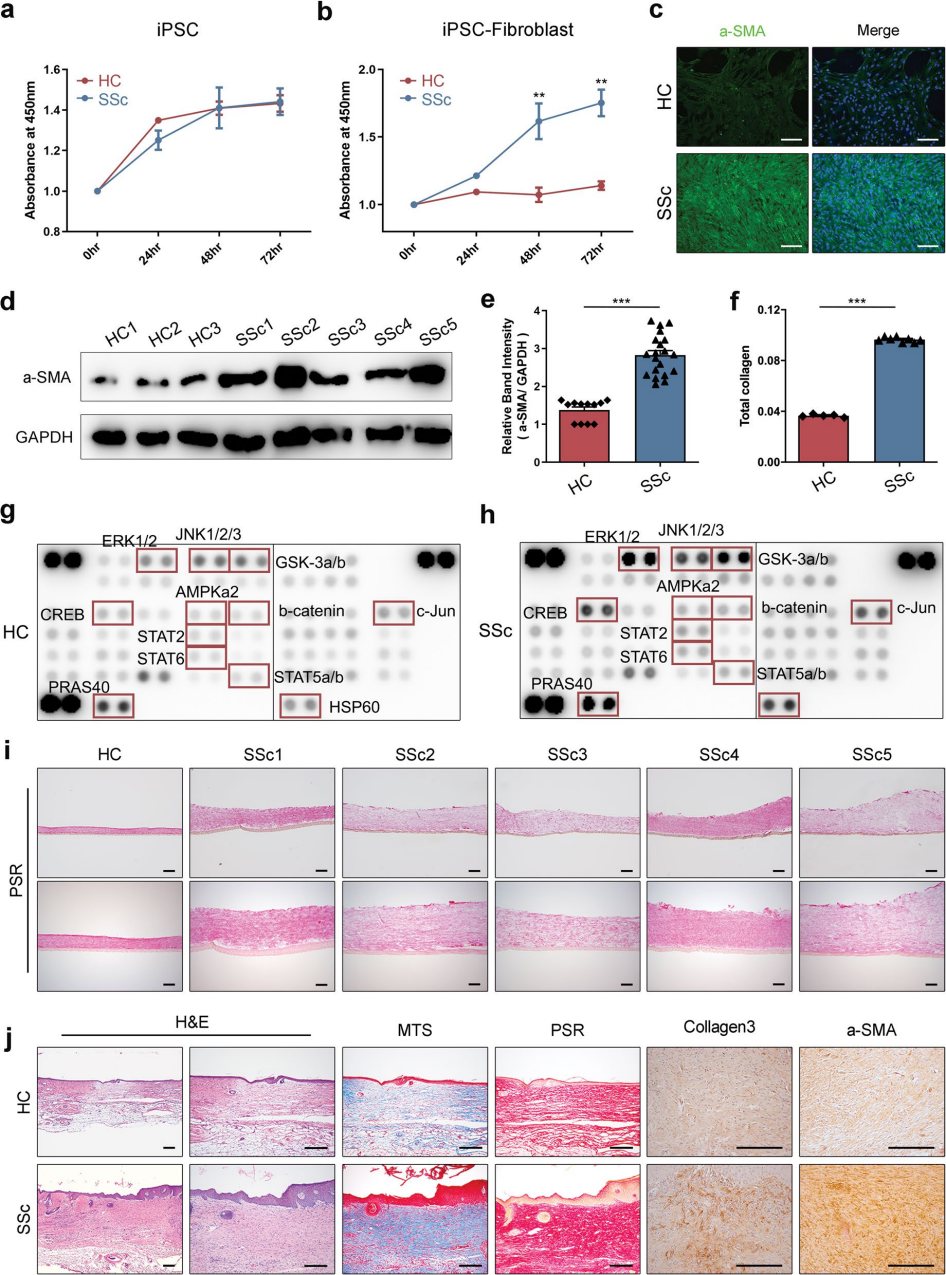
In vitro and in vivo modeling of systemic sclerosis using the induced pluripotent stem cells (iPSCs). **a**, **b** Proliferation assay of iPSCs (**a**) and their differentiation into fibroblasts (**b**). **c** Immunocytochemical analysis of α-SMA expression (green), together with DAPI staining was also performed (blue). Scale bars, 200 μm. **d** Expression of the fibrosis marker α-SMA, as determined by western blot. **e** Quantification of the relative band intensity by using imageJ. **f** Quantification of the total collagen contents by using the hydroxyproline assay. **g** Proteome profiler human phospho-kinase array of HC iPSC-derived fibroblast. **h** Proteome profiler human phospho-kinase array of SSc iPSC-derived fibroblast. **i** Histological analysis of the organoids and quantification of their culture area and thickness. Scale bars, 200 μm. **j** Histological analysis of the skin thickness and area of the transplanted organoids after 2 weeks. All graphs show the mean and standard error of the mean

The iPSC lines from the SSc patients and the healthy controls did not differ in terms of proliferation (Fig. 3a). After differentiation, however, the fibroblasts derived from the SSc iPSC lines proliferated more rapidly than the equivalent cells from the fibroblast derived from healthy-control iPSC lines (Fig. 3b). The SSc iPSC-derived fibroblasts also expressed more alpha smooth muscle actin(α-SMA) protein (Fig. 3c–e) and had higher total collagen concentrations (Fig. 3f). Thus, the iPSC-derived fibroblasts from SSc patients recapitulated the fibrotic phenotype of SSc fibroblasts. Analyzing the phosphorylation profiles of kinase and their protein substrates is essential for understanding how cells recognize and respond to changes in their environment. Severe fibrotic tissues from patients had increased levels of phosphorylation [28–34]. The SSc iPSC-derived fibroblasts increased phosphorylated protein than HC iPSC-derived fibroblasts. In particular, the phosphorylation was increased in ERK1/2, GSK-3a/b, CREB, c-Jun, PRAS40, and HSP60 (Fig. 3g, h). Furthermore, the iPSC-derived fibroblasts were cultured to produce three-dimensional (3D) fibroblast layer as dermal equivalent. The SSc-derived fibroblast layers were thicker, had a greater surface area, and had more cells than the healthy-control organoids (Fig. 3i). Thus, the SSc patient-derived 3D fibroblast layer recapitulated the fibrotic phenotype of the skin in SSc.

The iPSC-derived fibroblasts and keratinocytes were cultured together to produce three-dimensional skin organoids. We asked whether the SSc patient-derived organoids continued to exhibit accelerated fibrosis when they were xenografted onto immunodeficient mice. Thus, the organoids from the SSc patients and healthy controls were transplanted in the dorsal skin of NOD/SCID mice. After 2 weeks, the mice were killed, and the organoids were subjected to histology. Indeed, the SSc patient-derived organoids transplanted mice skin were thicker and had greater surface areas than the healthy control-derived organoids (Fig. 3j). The expression of collagen3 and α-SMA, fibrosis marker, was increased in SSc iPSC-derived skin organoid transplanted mice than health-control (Fig. 3j). Therefore, the SSc patient-derived skin organoids reflect the skin pathology of SSc in vitro and in vivo.

### FDA-approved drug library screening using iPSC-derived disease model

**Fig. 4:**
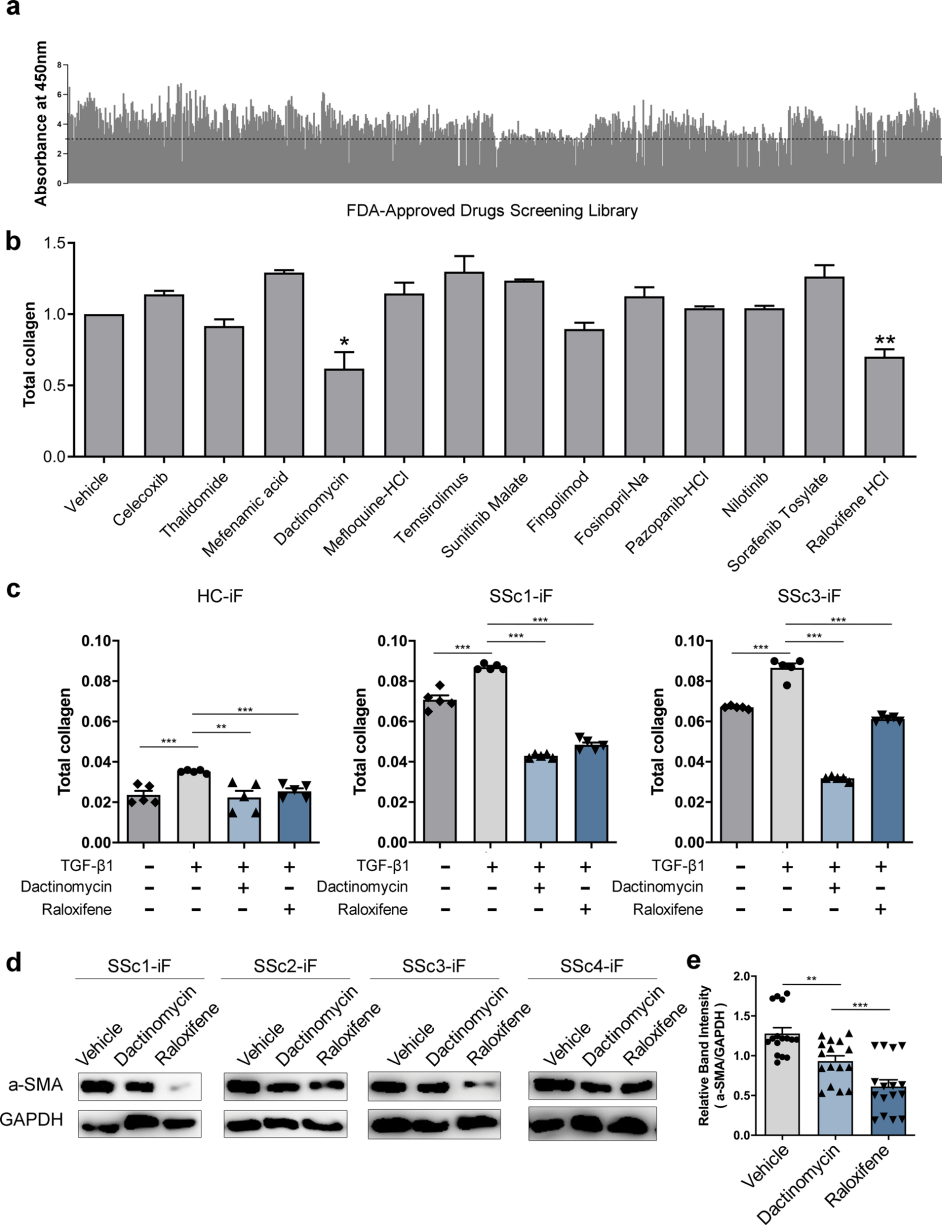
Screening of the FDA-approved drug library for an agent that reduces the accelerated fibrosis of induced pluripotent stem cells (iPSCs)-derived fibroblasts from systemic sclerosis patients. **a** Primary screen searching for drugs that reduced iPSC-derived fibroblast proliferation, as measured by using the Cell Counting Kit-8 assay. **b** Secondary screen of the drugs selected in primary screening. The ability of the drugs to reduce the total collagen content of the iPSC-derived fibroblasts was examined by using the hydroxyproline assay. **c** The ability of TGF-β1 to increase the total collagen levels of iPSC-derived fibroblasts, and the ability of dactinomycin and raloxifene to reduce this augmenting ability of TGF-β1, was examined by using the hydroxyproline assay. **d**, **e** The effect of TGF-β1 treatment with dactinomycin or raloxifene on the α-SMA expression of iPSC-derived fibroblasts was examined by western blot analysis. All graphs show the mean and standard error of the mean (*p < 0.05, **p < 0.01, ***p < 0.001, as determined by Student’s *t *test)

The most prominent feature of SSc is elevated fibroblast proliferation [23, 35]. The ability of 770 FDA-approved drugs to reduce the proliferation of iPSC-derived fibroblasts from the SSc patients was examined by using the Cell Counting Kit-8 (CCK-8) assay. In total, 48 drugs reduce the proliferation of SSc iPSC-derived fibroblasts (Fig. 4a).

Since fibroblasts from SSc patients also express higher collagen levels [36–38], we subjected the 48 selected drugs to a second screen for their ability to reduce the total collagen content of the iPSC-derived fibroblasts from the SSc patients. The hydroxyproline assay showed that 13 of the 48 drugs had this effect (Fig. 4b). Two particularly effective drugs were dactinomycin and raloxifene.

Transforming-growth factor (TGF)-β1 plays a major augmenting role in fibrosis [39–41]. Indeed, when we treated the iPSC-derived fibroblasts from SSc patients and healthy controls with TGF-β1, their total collagen content rose significantly. However, co-treatment with dactinomycin or raloxifene abrogated the ability of TGF-β1 to augment the total collagen levels in the fibroblasts (Fig. 4c).

Finally, the fibrosis in SSc associates with upregulated α-SMA expression in fibroblasts [42, 43]. When the iPSC-derived fibroblasts from the SSc patients were treated with raloxifene, their elevated α-SMA expression dropped. Dactinomycin was not more effective than raloxifene in reducing α-SMA expression (Fig. 4d, e). Therefore, we selected raloxifene as the hit drug for further analysis.

### Anti-fibrotic effect of raloxifene in vitro

**Fig. 5:**
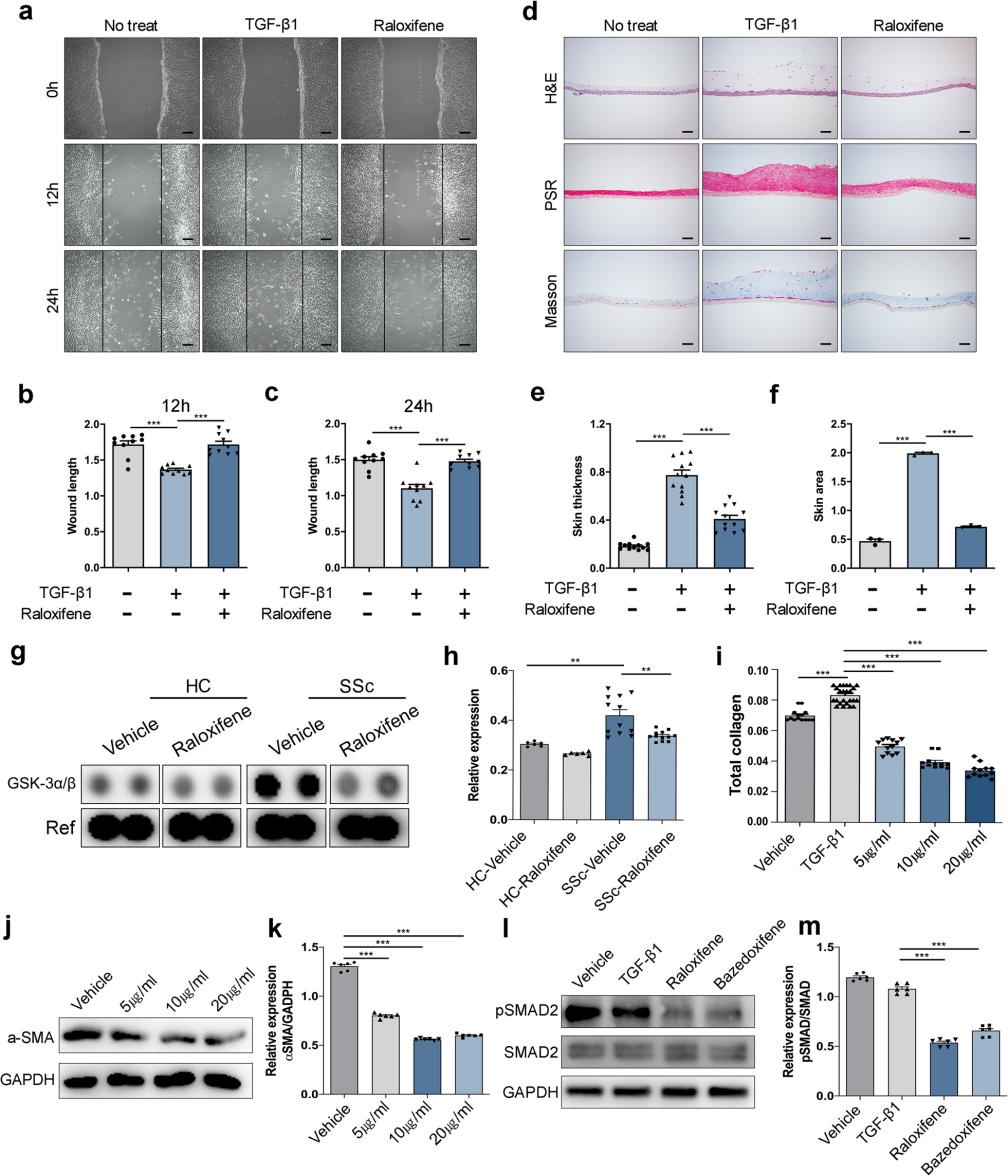
Anti-fibrotic effects of raloxifene in vitro. **a**–**c** The iPSC-derived fibroblasts from SSc patients were subjected to the scratch assay and the wound length was measured 12 (**b**) and 24 (**c**) hours later. Scale bars, 100 μm. **d**–**f** The three-dimensional dermal equivalent derived from SSc patients were treated with TGF-β1 in the presence or absence of raloxifene and the skin thickness and area were determined by histology. Scale bars, 100 μm. **g** Proteome profiler human phospho-kinase array of GSK-3α/β with treatment raloxifene. **h** Quantification of the relative intensity by using imageJ. **i** Total collagen concentration with various concentrations of raloxifene was assessed by the hydroxyproline assay. **j** α-SMA expression with various concentrations of raloxifene was assessed by western blot analysis. **k** Quantification of the relative band intensity by using imageJ. **l** Expression of phosphorylated SMAD2 signaling with treatment raloxifene and bazedoxifene in iPSC-Fs by western blot assay. **m** Quantification of the relative band intensity by using imageJ. All graphs show the mean and standard error of the mean (*p < 0.05, **p < 0.01, ***p < 0.001, as determined by Student’s *t *test)

To confirm that raloxifene has anti-fibrotic effects, the iPSC-derived fibroblasts were expanded to confluency, treated with TGF-β1 with or without raloxifene, and scratched. The wound length was measured 12 and 24 h later as an estimate of proliferation. Indeed, the TGF-β1-augmented proliferation of the fibroblasts was significantly reduced when raloxifene was also present (Fig. 5a–c). When the three-dimensional iPSC-derived fibroblast layers were treated with TGF-β1, the skin thickness increased. Treatment with raloxifene decreased this effect of TGF-β1 (Fig. 5d–f). Analyzing the phosphorylation profiles of kinase with treatment of raloxifene, the phosphorylation was decreased in GSK-3a/b (Fig. 5g, h). Similarly, when iPSC-derived fibroblasts from SSc patients were treated with various concentrations of raloxifene. This treatment also decreased the total collagen concentration in the cells in a raloxifene dose-dependent manner (Fig. 5i). Expression level of α-SMA dropped in a concentration-dependent manner (Fig. 5j, k). Raloxifene belongs to a class of selective estrogen receptor modulators (SERMs) that, depending on the target tissue, can act on the estrogen receptor as either an agonist or an antagonist [44]. SERMs include tamoxifene, raloxifene, lasofoxifene, bazedoxifene, and clomiphene citrate. Bazedoxifene has been shown to be relatively safe and well tolerated. To confirm SERMs has anti-fibrotic effects, raloxifene or bazedoxifene was treated iPSC-derived fibroblasts. Also, TGF-β1/SMAD signaling plays an important role in the pathogenesis of SSc [45]. The relative expression ratio of pSMAD2/SMAD2 was increased by treatment TGF-β1. By treating raloxifene or bazedoxifene, the expression of phosphorylated SMAD2 was downregulated (Fig. 5l, m). Thus, raloxifene could reduce the proliferation and fibrotic-factor expression of iPSC-derived fibroblasts in a dose-dependent manner. This suggests that raloxifene could have anti-fibrotic effects in iPSC-derived SSc model.

Furthermore, to confirm that raloxifene had anti-fibrotic effect in primary cells, health control and patient-derived fibroblasts were collected and expended. When the patient-derived fibroblasts were treated with bazedoxifene or raloxifene, their morphology was not changed (Additional file 2: Fig. S2a). Bazedoxifene and raloxifene reduce the proliferation of primary fibroblast (Additional file 2: Fig. S2b). The gene expression of fibrosis markers was increased in SSc fibroblast than health-control (Additional file 2: Fig. S2c, d). By treating bazedoxifene or raloxifene, wound length was reduced (Additional file 2: Fig. S2e–g) and total collagen concentration in the cells was also decreased than vehicle (Additional file 2: Fig. S2h). Similarly, when the three-dimensional primary fibroblast layers were treated with bazedoxifene or raloxifene. Raloxifene was more effective than bazedoxifene in reducing thickness of 3D fibroblast layers (Additional file 2: Fig. S2i–k). Total collagen dropped in a concentration-dependent manner with various concentrations of raloxifene (Additional file 2: Fig. S2l). This suggests that raloxifene could have anti-fibrotic effects in patient-derived primary fibroblasts.

### Anti-fibrotic effect of raloxifene in a mice model of SSc

**Fig. 6:**
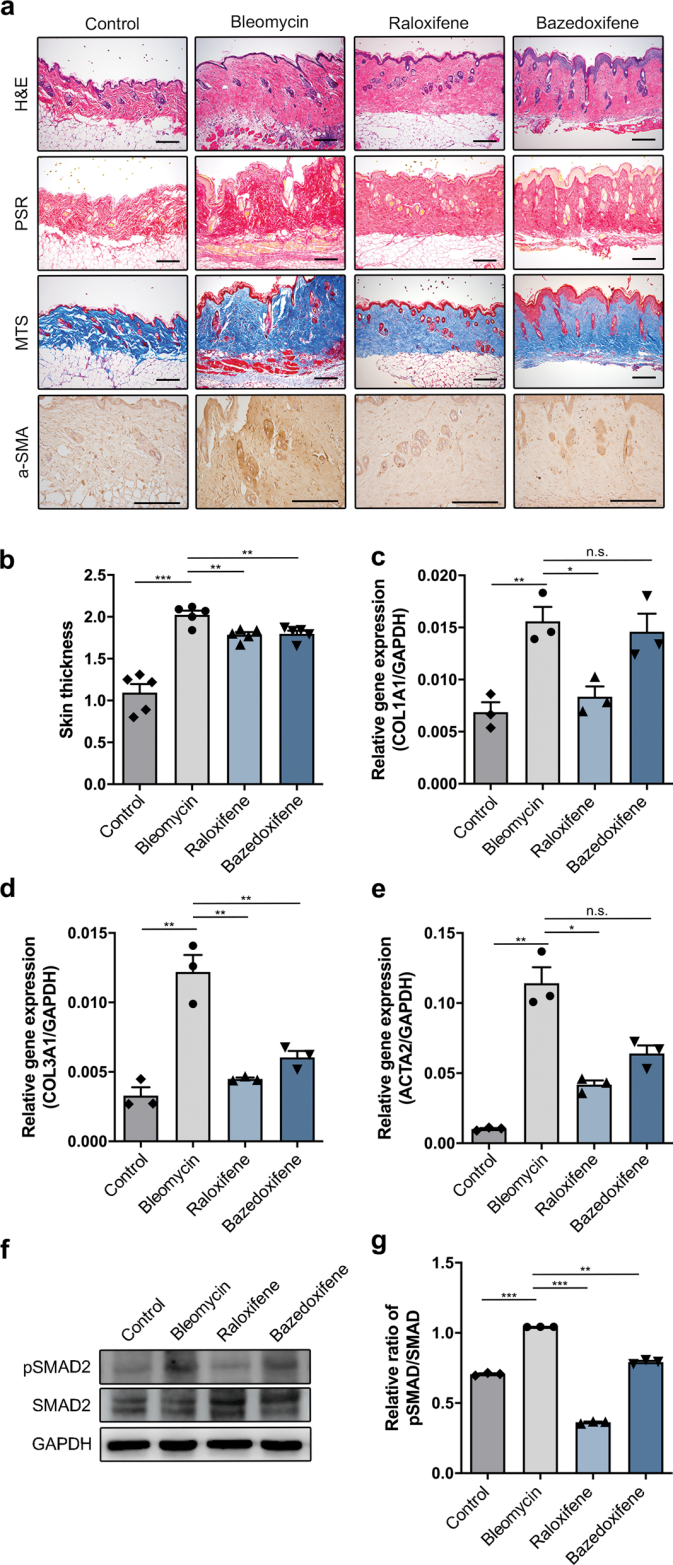
Anti-fibrotic effects of raloxifene in the bleomycin-induced model of systemic sclerosis. **a** Their dorsal skin thickness was examined by histology, Scale bars, 100 μm. **b** Quantification of the skin thickness by histology of the bleomycin induction model. **c-e** Expression of the fibrosis marker genes of COL1A1, COL3A1 and ACTA2 in bleomycin model. **f**, **g** After 3 weeks of raloxifene treatment, bleomycin mice skin expression of pSMAD was assessed by western blot analysis. All graphs show the mean and standard error of the mean (*p < 0.05, **p < 0.01, ***p < 0.001, as determined by Student’s *t *test)

The bleomycin-induced mice model is a commonly used chemical model of SSc [46, 47]. Therefore, to confirm that raloxifene has anti-fibrotic effects in mice model of SSc, we generated the bleomycin-induced model of SSc by daily subcutaneous bleomycin injections. Starting 3 days later, the mice were also treated with daily subcutaneous injections of raloxifene. Raloxifene is anti-osteoporotic drug and used for the treatment prevention of osteoporosis in postmenopausal women. Raloxifene is a selective estrogen receptor modulator (SERM). We treated bazedoxifene, which is a kind of SERM-class drug in bleomycin mice model. After 3 weeks, the mice were killed, and their skin thickness was examined by histology (Fig. 6a, b). Also, the gene expression of fibrosis markers COL1A1, COL3A1 and ACTA2 was increased by bleomycin induction and this effect was attenuated by raloxifene treatment (Fig. 6c–e). Their skin expression of pSMAD was also assessed by western blot analysis. The expression level of pSMAD2/SMAD2 was increased by treatment bleomycin. By treating raloxifene, the expression was downregulated (Fig. 6f, g).

Many variables of fibrosis marker were increased by the bleomycin injections but the raloxifene injections significantly reduced these effects. Therefore, these results suggested that raloxifene therapy had anti-fibrotic effects in the bleomycin-induced model of SSc.

## Discussion

Fibrosis is a pathological symptom of chronic inflammatory disease. Fibrosis, or scarring, is determined by the accumulation of excess extracellular matrix component (ECM) such as collagen and fibronectin [48, 49]. Fibrosis becomes dysregulated following tissue injury. When tissues are injured, fibroblast becomes activated, increasing their contractility, secretion of inflammatory cytokines, and synthesis of ECM components. Fibroblasts are the common cell type of the connective tissues and the major source of the ECM. Also, fibroblasts are the effective mediator of the pathological fibrotic accumulation of ECM, proliferation and differentiation that results in tissue injury and chronic inflammation [50]. Skin fibrosis occurs locally in response to dermal injury according to burn, surgery, trauma, infection, or radiation, or in association with systemic diseases such as scleroderma and graft-versus-host disease [51–53]. Scleroderma (SSc) is an autoimmune disease associated with high morbidity and mortality [51, 54]. SSc is heterogeneous and rare disease, and the pathogenesis is characterized by three hallmarks of small vessel vasculopathy, autoantibodies, and fibroblast dysfunction resulting in increased deposition of extracellular matrix [1, 2]. In SSc, fibrosis occurs in the skin and progress to many organs, including esophagus, gastrointestinal track, lung, kidney, heart [3, 4]. Fibrosis is the result of activation of fibroblast and deposition of excessive ECM, which are critical features of SSc [55]. Development of effective treatment of SSc has been hampered by a lack of sufficient understanding of its pathophysiology, partly because of its heterogeneity and partly because it is an orphan disease and patient-derived biomaterials are scarce [6, 7].

Recently, a promising strategy for overcoming the biomaterial scarcity in orphan diseases is to generate induced pluripotent stem cells (iPSCs) [56–58]. The present study showed that the paucity of biomaterials in dermatology can be overcome by generating iPSCs from PBMCs of patients and inducing them to differentiate into skin cells, particularly the fibroblasts, which play a key role in the pathophysiology [23, 35, 43]. We observed that the fibroblasts derived from iPSCs of SSc patients exhibited significantly greater proliferation and produced more ECM than the equivalent fibroblasts from healthy-control subjects. Severe fibrotic tissues from patients had increased levels of phosphorylation [28–34]. Phosphorylation of fibrosis related proteins was increased SSc iPSC-derived fibroblasts than HC iPSC-derived fibroblast. Moreover, when they were used together with keratinocytes derived from the same iPSC lines to generate skin organoids, the organoids from the SSc patients were thicker and larger than those from the healthy controls. In addition, when these organoids were transplanted into the dorsal skin of immunodeficient mice, they were thicker than the healthy-control organoids. Thus, fibroblasts derived from SSc iPSCs bear the same pathological features as fibrosis of SSc patients. This suggests that these cells may be useful for studying the pathological mechanism underlying SSc and for identifying effective therapies. This notion is supported by the fact that SSc is not a Mendelian genetic disorder: environmental changes and the resulting epigenetic modifications can be important factors that drive the development of this disease [59, 60]. Moreover, it has been shown that since epigenetic modifications can remain after somatic cell reprogramming, cells that are differentiated from the iPSCs of somatic cells can reflect the disease phenotypes that lead to diseases such as cardiomyopathy and neuronal disease [61, 62].

In recent years, considerable effort has gone into identifying anti-fibrotic therapies [14]. Nevertheless, only a few therapies that can suppress fibrosis have been discovered [63]. One may be mesenchymal stem cell (MSC) therapy. However, the anti-fibrotic effects of MSC are either small or only effective in the early stage of fibrosis [64]. Human cells that are derived from iPSCs are increasingly being used as high-throughput platforms for the screening of diverse compounds [9, 10, 65]. We used this approach to identify drugs that can suppress the pro-fibrotic activity of SSc fibroblasts. Thus, iPSC-derived fibroblasts from SSc patients were tested with the FDA-approved drug library, which consists of 770 drugs. The first screen showed that 48 drugs effectively suppressed SSc-fibroblast proliferation. The second screen then showed that 13 drugs efficiently reduced not only SSc-fibroblast proliferation but also ECM protein synthesis. Interestingly, of these 13 drugs, raloxifene was the most effective anti-fibrotic agent. Raloxifene belongs to a class of selective estrogen receptor modulators (SERMs) that, depending on the target tissue, can act on the estrogen receptor as either an agonist or an antagonist [44]. Raloxifene has strong anti-estrogenic effects and is thus a very well-known and effective anti-osteoporotic drug [66]. Two of the most common SERMs are tamoxifene and raloxifene. There are several others as well, including lasofoxifene, bazedoxifene, and clomiphene citrate. Bazedoxifene has been shown to be relatively safe and well tolerated. SERMs are competitive partial agonist of the ER. Different tissues have different degrees of sensitivity to the activity of endogenous estrogens, so SERMs produce estrogenic or anti-estrogenic effects depending in the specific tissue in question as well as the percentage of intrinsic activity (IA) of the SERM [67]. Furthermore, affinities of estrogen receptor ligands for the ERα and EBβ are used depending on the type of SERMs [68–71]. The effects and affinities acting on the skin are different; it is expected that the effects will also be different depending on the type of SERMs.

In this study, we suggest that the anti-estrogen drug of raloxifene has anti-fibrotic properties. As mentioned above, it reduced the excessive proliferation and ECM production of iPSC-derived fibroblasts from SSc patients. It also reversed the pro-fibrotic effects of TGF-β treatment on these cells. This cytokine participates in a pathway with SMAD that converts SSc fibroblasts into myofibroblasts [39–41]. Since these cells proliferate strongly and produce high levels of α-SMA and ECM proteins (e.g., collagen types I and III, vimentin, and fibronectin), the TGF-β1/SMAD pathway plays an important role in the pathogenesis of SSc [45]. Also, Wnt signaling was aberrantly activated and produced of TGF-β1 in fibrosis tissues. GSK-3 is a key factor as glycogen synthase kinase in Wnt signaling. The phosphorylation of GSK-3 can diminish the activity of GSK-3 and activate β-catenin. Thus, we observed that while TGF-β1 treatment increased the thickness of SSc-derived skin organoids and the ECM production, α-SMA expression, and proliferation of SSc iPSC-derived fibroblasts, treatment of raloxifene decreased all these effects of TGF-β1. TGF-β1 treatment increased pSMAD2 expression of SSc iPSC-derived fibroblast that was decreased by raloxifene treatment. Furthermore, raloxifene reduced the phosphorylation of GSK-3. To confirm that raloxifene has anti-fibrotic effects in SSc, we generated the bleomycin-induced murine model of SSc and injected the mice with raloxifene. Raloxifene effectively reduced the skin thickness of the mice along with the expression by the skin of the fibrotic-marker genes COL1A1, COL3A1, and ACTA2. Furthermore, bleomycin increased pSMAD2 expression of mice skin that was decreased by raloxifene treatment. Therefore, this result suggested that the GSK-3β and TGF-β1/Smad2/3 signaling may be important pathways as antifibrotic activity of raloxifene.

## Conclusions

In conclusion, raloxifene reduced SSc-related fibrosis by downregulating fibroblast proliferation and ECM production. This is a clinically important observation because raloxifene is a very safe and well-tolerated anti-osteoporotic drug and our data suggest that it may be useful for intractable fibrosis in SSc. We also observed that a closely related SERM, bazedoxifene, had a similar anti-fibrotic effect. Thus, SERM-class drugs might be candidate therapeutic drugs for SSc in the near further.

## Supplementary Information

**Additional file 1: Figure S1**. Characterization of induced pluripotent stem cells (iPSCs) from patients with systemic sclerosis (SSc) and healthy controls. **a** Morphology of iPSCs, as determined by Leica microscopy. Scale bars, 200 μm. **b** Alkaline phosphatase staining of iPSCs. Scale bars, 200 μm. **c** Immunocytochemical analysis of iPSC expression of the pluripotency-marker proteins Oct4, SSEA4, Sox2, TRA-1–60, Klf4, and TRA-1–81. Scale bars, 200 μm. **d** RT-PCR analysis of iPSC expression of the pluripotency-marker genes OCT4, SOX2, NANOG, and LIN28. All graphs show the mean and standard error of the mean (*p < 0.05, **p < 0.01, ***p < 0.001, as determined by Student’s *t *test).

**Additional file 2: Figure S2**. Anti-fibrotic effects of raloxifene in systemic sclerosis (SSc) patient-derived primary fibroblasts. **a** Morphology of primary fibroblast with bazedoxifene and raloxifene treatment, as determined by Leica microscopy. Scale bars, 100 μm. **b** Proliferation assay of primary fibroblasts with bazedoxifene and raloxifene treatment. **c**, **d** RT-PCR analysis of fibrosis marker genes COL1A1, and ACTA2. **e** HC and Patient-derived fibroblasts were subjected to the scratch assay with bazedoxifene and raloxifene treatment and the wound length was measured 24 h later. Scale bars, 100 μm. **f**, **g** Quantification of the wound length by using imageJ. **h** Total collagen concentration with bazedoxifene and raloxifene was assessed by the hydroxyproline assay. **i**–**k** The three-dimensional dermal equivalent derived from primary fibroblasts were treated with bazedoxifene and raloxifene and the skin thickness were determined by histology. Scale bars, 100 μm. **l** Total collagen concentration with various concentrations of raloxifene was assessed by the hydroxyproline assay. All graphs show the mean and standard error of the mean (*p < 0.05, **p < 0.01, ***p < 0.001, as determined by Student’s *t *test).

## Data Availability

All datasets of this article are included within the article.

## Abbreviations

α-SMA: Alpha smooth muscle actin
BMP4: Bone morphogenetic protein 4
EB: Embryonic body
ECM: Extracellular matrix
EGF: Epidermal growth factor
ESC: Embryonic stem cell
iF: IPSC-derived fibroblast
iK: IPSC-derived keratinocyte
iPSC: Induced pluripotent stem cell
iSO: Induced pluripotent stem cell derived skin organoid
MTS: Masson’s trichrome staining
PBMC: Peripheral blood mononuclear cell
PSR: Picrosirius red
RA: Retinoic acid
SERM: Selective estrogen receptor modulator
SSc: Systemic sclerosis
TGF: Transforming growth factor
